# Partial Genetic Deletion of Klotho Aggravates Cardiac Calcium Mishandling in Acute Kidney Injury

**DOI:** 10.3390/ijms24021322

**Published:** 2023-01-10

**Authors:** Laura González-Lafuente, José Alberto Navarro-García, Ángela Valero-Almazán, Elena Rodríguez-Sánchez, Sara Vázquez-Sánchez, Elisa Mercado-García, Patricia Pineros, Jonay Poveda, María Fernández-Velasco, Makoto Kuro-O, Luis M. Ruilope, Gema Ruiz-Hurtado

**Affiliations:** 1 Cardiorenal Translational Laboratory, Institute of Research Imas12, Hospital Universitario 12 de Octubre, 28041 Madrid, Spain; 2 IdiPAZ Institute for Health Research/Centro de Investigación Biomédica en Red de Enfermedades Cardiovasculares, CIBER-CV, 28046 Madrid, Spain; 3 Division of Anti-Ageing Medicine, Centre for Molecular Medicine, Jichi Medical University, Shimotsuke 329-0498, Japan; 4 Centro de Investigación Biomédica en Red de Enfermedades Cardiovasculares (CIBER-CV), Hospital Universitario 12 de Octubre, 28041 Madrid, Spain; 5 School of Doctoral Studies and Research, European University of Madrid, 28040 Madrid, Spain; 6 Departamento de Fisiología, Facultad de Medicina, Universidad Autónoma de Madrid, 28029 Madrid, Spain

**Keywords:** klotho, acute kidney injury, cardiorenal syndrome, cardiomyocyte, calcium handling, arrhythmia

## Abstract

Acute kidney injury (AKI) is associated with an elevated risk of cardiovascular major events and mortality. The pathophysiological mechanisms underlying the complex cardiorenal network interaction remain unresolved. It is known that the presence of AKI and its evolution are significantly associated with an alteration in the anti-aging factor klotho expression. However, it is unknown whether a klotho deficiency might aggravate cardiac damage after AKI. We examined intracellular calcium (Ca^2+^) handling in native ventricular isolated cardiomyocytes from wild-type (+/+) and heterozygous hypomorphic mice for the klotho gene (+/*kl*) in which an overdose of folic acid was administered to induce AKI. Twenty-four hours after AKI induction, cardiomyocyte contraction was decreased in mice with the partial deletion of klotho expression (heterozygous hypomorphic klotho named *+/kl*). This was accompanied by alterations in Ca^2+^ transients during systole and an impairment of sarco/endoplasmic reticulum Ca^2+^-ATPase (SERCA2a) function in *+/kl* mice after AKI induction. Moreover, Ca^2+^ spark frequency and the incidence of Ca^2+^ pro-arrhythmic events were greater in cardiomyocytes from heterozygous hypomorphic klotho compared to wild-type mice after AKI. A decrease in klotho expression plays a role in cardiorenal damage aggravating cardiac Ca^2+^ mishandling after an AKI, providing the basis for future targeted approaches directed to control klotho expression as novel therapeutic strategies to reduce the cardiac burden that affects AKI patients.

## 1. Introduction

Klotho is an aging suppressor gene that encodes a single-pass transmembrane protein expressed in multiple tissues, with the highest expression in the renal tubules, parathyroid glands and choroid plexus of the brain [1,2]. Klotho was originally identified in a strain of mutant mice that was unable to express klotho and exhibited premature aging, shortened lifespan and the development of multiple aging disorders [1]. In addition to its transmembrane form, klotho can be cleaved by anchored proteases in the extracellular medium and released into the systemic circulation, giving rise to its soluble form. Soluble klotho exerts its actions in an autocrine and paracrine manner in different target tissues [3,4]. Membrane klotho serves as a co-receptor for fibroblast growth factor 23 (FGF23) and regulates phosphorus and vitamin D metabolism, which are essential for endogenous phosphaturic FGF23 actions [5]. The soluble form of klotho has been described to exhibit several systemic beneficial effects such as anti-inflammatory, antioxidant, antifibrotic and antiapoptotic actions [6,7,8]. It has been demonstrated that serum levels of klotho decrease with aging and it is also associated with several typical age-related disorders such as diabetes, vascular calcification, atherosclerosis, cardiovascular and renal diseases [4,6,9]. In the kidney, low circulating plasma soluble klotho levels have been correlated with worsening renal function in chronic kidney disease (CKD) and end-stage renal disease (ESRD) as well as in acute kidney injury (AKI) [10]. Furthermore, low soluble klotho levels are associated with arterial stiffness in CKD, increased cardiovascular mortality rate in haemodialysis and cardiovascular disease risk factors in older adults [11,12,13].

Cardiorenal syndrome (CRS) is defined as a complex pathological condition in which kidney and heart function is impaired, whereby acute or chronic dysfunction in one organ can induce acute or chronic dysfunction in the other [14]. This syndrome, in any of its forms, is clinically relevant as the coexistence of cardiovascular and renal disease significantly increases the risk of major cardiovascular events, such as congestive heart failure and even mortality [15,16]. Depending on the origin of the pathology, CRS can be classified into five different subtypes. Specifically, CRS type 3 or acute reno-cardiac syndrome consists of AKI leading to acute cardiac alterations [17]. Recently, it has been demonstrated that several uremic experimental models develop a cardiac failure phenotype with relevant cardiomyocyte alterations including contractile dysfunction and intracellular calcium (Ca^2+^) mishandling [18,19,20,21]. In this uremic cardiomyopathy setting, klotho has emerged as a new therapeutic target to protect cardiac function by normalising intracellular Ca^2+^ handling in ventricular cardiomyocytes, where endogenous klotho overexpression or increasing its bioavailability exogenously through recombinant murine klotho administration improves cardiomyocyte function and impedes deleterious cardiac remodelling associated with renal damage [20,22,23,24]. On the other hand, a severe klotho deficiency, as happens in hypomorphic *kl/kl* mice, significantly worsens cardiomyocyte function, inducing a deleterious ionic remodelling that predisposes them to suffer heart failure and fatal ventricular arrhythmias even in the absence of any additional renal insult [18,24]. However, it remains unclear whether a moderate decrease in klotho expression could predispose a cardiac outcome after renal damage. Thus, the aim of this study was to investigate whether a mild deficiency in klotho expression due to the partial deletion of its endogenous expression in heterozygous *+/kl* mice worsens cardiomyocyte function after AKI.

## 2. Results

### 2.1. Macroscopic and Biochemical Parameters of Renal Function in +/+ and +/kl Mice after AKI

Macroscopic and biochemical parameters were analysed to assess macroscopic renal and cardiac damage associated with folic acid (FA)-induced AKI, and whether they could be aggravated by klotho deficiency (Table 1 and Table 2). AKI mice showed no significant differences in body weight (BW) or tibia length (TL) between experimental groups. Kidney weight (KW) and KW/body weight (KW/BW) ratio were significantly higher in mice with AKI, regardless of genotype. This increase was also observed when KW/tibia length ratio (KW/TL) was analysed (*p* < 0.05 for +/*kl*-FA vs. +/*kl* mice). Increased KW indicates renal hypertrophy, which is probably a compensatory mechanism for the renal damage caused by FA crystallisation in renal tubules. We did not observe significant differences in heart weight (HW), HW/BW or HW/TL in any of the experimental groups.

The results related to renal damage are shown in Table 2. Urea and blood urea nitrogen (BUN) were significantly higher in the FA-treated groups (*p* < 0.001) relative to each control group independent of genotype. FA-treated wild-type (+/+) mice had higher phosphate concentrations than its control group (*p* < 0.001). Similarly, heterozygous hypomorphic klotho (+/*kl*) mice treated with FA showed higher phosphate levels than its control group, with the +/*kl* mice (*p* < 0.001) being significantly lower than wild-type +/+ FA-mice (*p* < 0.001). In FA-treated mice, there was a significant increase in plasma FGF23 levels independently of the genotype (*p* < 0.001 vs. +/+ and +/*kl*), although +/*kl*-FA mice showed significantly lower FGF23 levels than +/+-FA mice.

Furthermore, to confirm kidney damage in our experimental model, we measured the expression levels of kidney injury molecule-1 (KIM-1) and NGAL (neutrophil gelatinase-associated lipocalin), both considered kidney damage markers, as well as renal Klotho mRNA expression (Supplementary Figure S1). We found that both NGAL and KIM-1 expression was increased in FA-treated mice (*p* < 0.001 for KIM-1, and *p* < 0.01 for NGAL), with KIM-1 expression still higher in +/*kl*-FA compared to +/+-FA mice (*p* < 0.001). Klotho expression at the renal level was significantly decreased in AKI mice, regardless of genotype (*p* < 0.001 vs. +/+, and *p* < 0.01 vs. +/*kl*). All these data together (urea, BUN, FGF23, phosphates, NGAL, KIM-1 and klotho levels) clearly indicate that an FA-induced AKI model causes damage to the kidneys independently of the genotype of the mice.

### 2.2. Partial Deficiency in Klotho Expression Aggravates Cellular Contractile Dysfunction after AKI

**Figure 1:**
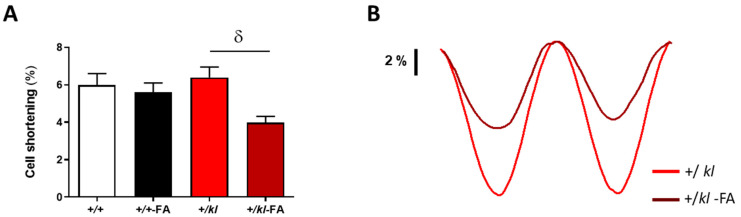
Partial klotho deficiency aggravates the cardiomyocyte contractile function after AKI. (**A**) Cell shortening of cardiomyocytes from wild-type +/+ (n = 48 cells, N = 5 mice), +/+-FA (n = 66 cells, N = 4 mice), +/*kl* (n = 46 cells, N = 5 mice) and +/*kl*-FA animals (n = 54 cells, N = 3 mice). (**B**) Shortening profiles examples of cardiomyocytes from +/*kl* and +/*kl*-FA mice. Data are expressed as mean ± SEM. ^δ^ *p* < 0.05 vs. +/*kl*.

We determined whether cardiomyocyte contractility was worsened after FA-induced AKI when klotho expression was reduced. In the absence of AKI, no significant differences in cell contraction were found depending on the genotype of the mice, being similar in +/+ and +/*kl* groups (Figure 1A). No significant differences were found in cardiomyocyte contraction in FA-treated wild-type +/+ mice compared to the +/+ vehicle group. However, cardiomyocyte contraction was reduced when AKI was induced in +/*kl* mice compared to the +/*kl* vehicle group (*p* < 0.05, Figure 1A). These data suggest that klotho expression is important to maintain contractile function in cardiomyocytes after AKI induction.

### 2.3. Klotho Deficiency Induces Alterations in Systolic Ca^2+^ Release after AKI

**Figure 2:**
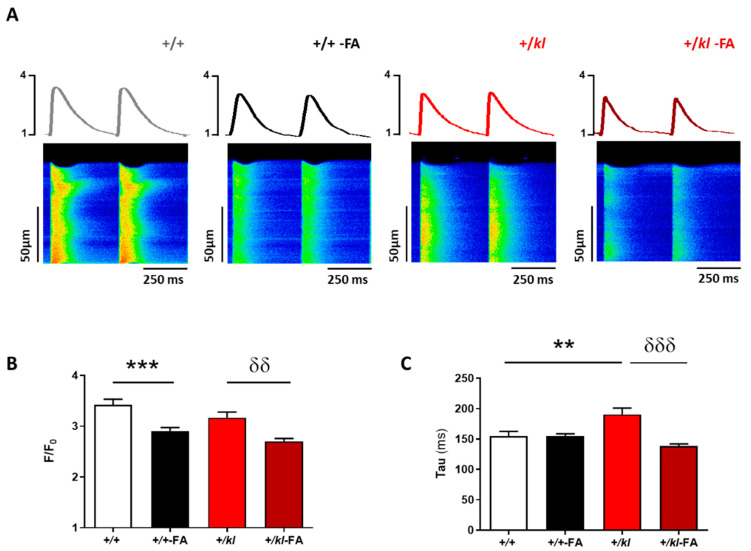
Partial klotho deficiency aggravates systolic Ca^2+^ release from sarcoplasmic reticulum (SR) after AKI. (**A**) Fluorescence profiles (upper panel) of the representative line-scan confocal images of Ca^2+^ transients (bottom panel). (**B**) Mean peak fluorescence of Ca^2+^ transients (F/F_0_). (**C**) Decay time constant, Tau (ms) of cardiomyocytes isolated from +/+ (n = 49 cells, N = 5 mice), +/+-FA (n = 81 cells, N = 4 mice), +/*kl* (n = 47 cells, N = 5 mice) and +/*kl*-FA animals (n = 66 cells, N = 3 mice). Data are expressed as mean ± SEM. ** *p* < 0.01, *** *p* < 0.001 vs. +/+, ^δδ^ *p* < 0.01, ^δδδ^ *p* < 0.001 vs. +/*kl*.

Due to the alterations found in cardiomyocyte contractile function and the close relationship between cardiac contractile dysfunction and alterations in intracellular Ca^2+^ handling, we analysed systolic Ca^2+^ release in isolated ventricular cardiomyocytes from AKI mice with klotho deficiency. Figure 2A shows the representative fluorescence profiles and line-scan Ca^2+^ images from Fluo-3AM-loaded cardiomyocytes during electrical stimulation at 2 Hz obtained by confocal microscopy of each experimental group. Image analysis revealed that cardiomyocytes isolated from FA-injected animals exhibited a significant decrease in the amplitude of the intracellular Ca^2+^ transients (measured as F/F_0_) compared to their corresponding controls (*p* < 0.001 vs. +/+ and *p* < 0.01 vs. +/*kl*, Figure 2B) independently of the genotype. The time constant of Ca^2+^ transient decay (Tau) in cardiomyocytes from the +/*kl* vehicle group was significantly higher compared with cardiomyocytes from +/+ vehicle animals (*p* < 0.01, Figure 2C). No differences in Tau were observed in cardiomyocytes between the +/+-vehicle and +/+-FA groups. However, in cardiomyocytes from +/*kl*-FA mice, Tau was significantly lower compared to those mice with the same genetic background without AKI induction (*p* < 0.001, Figure 2C). In addition, K SERCA2a, the SR-dependent contribution to the decay rate constant of the systolic Ca^2+^ transient, was also determined by subtracting the decay rate constant of the caffeine-triggered transients from that of the systolic Ca^2+^ transients. K SERCA2a was significantly higher in cardiomyocytes from +/*kl*-FA mice than in vehicle- +/*kl* mice (*p* < 0.05 vs. +/*kl*, Supplementary Figure S2), supporting an increased SERCA2a-mediated Ca^2+^ re-uptake in +/*kl*-FA mice.

### 2.4. AKI Reduces the SR-Ca^2+^ Load Independently of Klotho Availability

**Figure 3:**
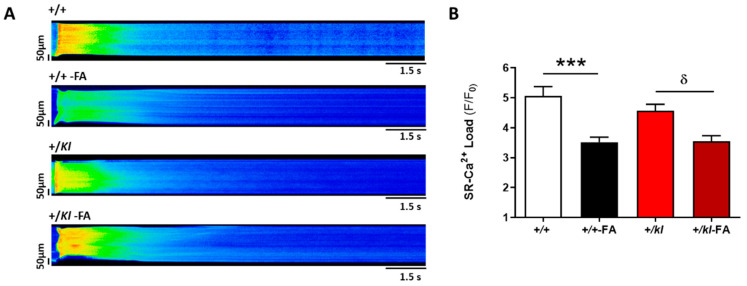
AKI decreases SR-Ca^2+^ load in adult ventricular cardiomyocytes independently of the klotho expression. (**A**) Representative line-scan images of caffeine-evoked Ca^2+^ transients in cardiomyocytes from each experimental group obtained by confocal microscopy. (**B**) Mean values of Ca^2+^ transient amplitude evoked by caffeine (peak fluorescence Ca^2+^, F/F_0_) in cardiomyocytes from +/+ (n = 24 cells, N = 5 mice), +/+-FA (n = 39 cells, N = 4 mice), +/*kl* (n = 27 cells, N = 5 mice) and +/*kl*-FA mice (n = 25 cells, N = 3 mice). Data are expressed as mean ± SEM. *** *p* < 0.001 vs. +/+, ^δ^ *p* < 0.05 vs. +/*kl*.

To determine whether the reduction in systolic Ca^2+^ release previously observed in +/+ and +/kl with AKI may be conditioned by alterations in SR Ca^2+^ load, we acutely perfused caffeine in ventricular cardiomyocytes to deplete SR Ca^2+^ stores. Figure 3A shows representative line-scan images of caffeine-evoked Ca^2+^ transients in each experimental group. Independently of genotype, the amplitude of Ca^2+^ transients induced by caffeine application were significantly lower in those animals with AKI compared to their corresponding controls (*p* < 0.001 vs. +/+ and *p* < 0.05 vs. +/*kl*, Figure 3B), indicating that partial klotho deficiency did not further decrease SR Ca^2+^ load to those observed under AKI conditions.

### 2.5. Klotho Deficiency Aggravates Diastolic Ca^2+^ Leak after AKI

**Figure 4:**
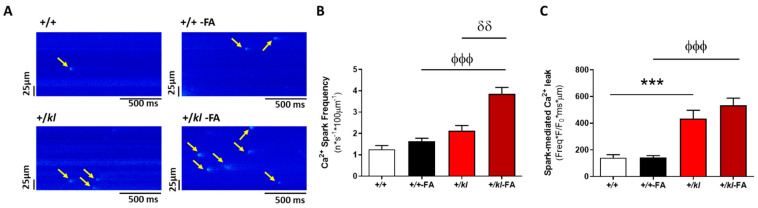
Partial klotho deficiency increases the frequency of diastolic Ca^2+^ leak in cardiomyocytes after AKI four times. (**A**) Representative line-scan images of Ca^2+^ sparks in quiescent cardiomyocytes from +/+, +/+-FA, +/*kl* and +/*kl*-FA mice. The arrows point to the released Ca^2+^ sparks in each representative image. (**B**) Frequency of Ca^2+^ sparks (presented in n·s^−1^·100 µm^−1^) in cardiomyocytes from +/+ (n = 49 cells, N = 5 mice), +/+-FA (n = 75 cells, N = 4 mice), +/*kl* (n = 48 cells, N = 5 mice) and +/*kl*-FA mice (n = 57 cells, N = 3 mice). (**C**) Spark-mediated Ca^2+^ leak: frequency × amplitude (F/F0) × duration (ms) × width (µm) of Ca^2+^ sparks in cardiomyocytes from +/+ (n = 49 cells, N = 5 mice), +/+-FA (n = 75 cells, N = 4 mice), +/*kl* (n = 48 cells, N = 5 mice) and +/*kl*-FA mice (n = 57 cells, N = 3 mice). Data are expressed as mean ± SEM. *** *p* < 0.001 vs. +/+, ^ϕϕϕ^ *p* < 0.001 vs. +/+-FA, ^δδ^ *p* < 0.01 vs. +/*kl*.

We next analysed the frequency of Ca^2+^ sparks as an indirect form to estimate the activity of ryanodine receptors (RyR_2_) and the SR Ca^2+^ leak during diastole. Figure 4A shows representative line-scan images of Ca^2+^ sparks in unstimulated cardiomyocytes from each experimental group. Under AKI conditions, cardiomyocytes from heterozygous +/*kl*-FA mice had a strong increase in frequency of Ca^2+^ sparks compared to those +/+ mice after AKI or +/*kl* mice in the absence of AKI (*p* < 0.001 vs. +/+, *p* < 0.01 vs. +/*kl*, Figure 4B). To evaluate the kinetic properties of RyR_2_ clusters, the biophysical characteristics of the Ca^2+^ sparks were analysed such as amplitude (measured as the peak F/F_0_), full duration at half maximum (FDHM) and full width at half maximum (FWHM) in cardiomyocytes from each experimental group (Supplementary Table S1). FA-induced kidney damage provoked a significant decrease in the Ca^2+^ spark amplitude and width, independently of the genotype of mice (*p* < 0.001, Supplementary Table S1); whereas Ca^2+^ spark duration was longer in vehicle-+/*kl* mice than in vehicle-treated +/+ mice (*p* < 0.001, Supplementary Table S1) and the duration of Ca^2+^ sparks was reduced in FA-treated +/*kl* mice compared to their control group (*p* < 0.001, Supplementary Table S1). The calculated spark-mediated Ca^2+^ leak from the SR was significantly increased in vehicle-treated +/*kl* compared to +/+ vehicle-treated mice (*p* < 0.001, Figure 4C) as well as between both groups with AKI (*p* < 0.001, Figure 4C).

**Figure 5:**
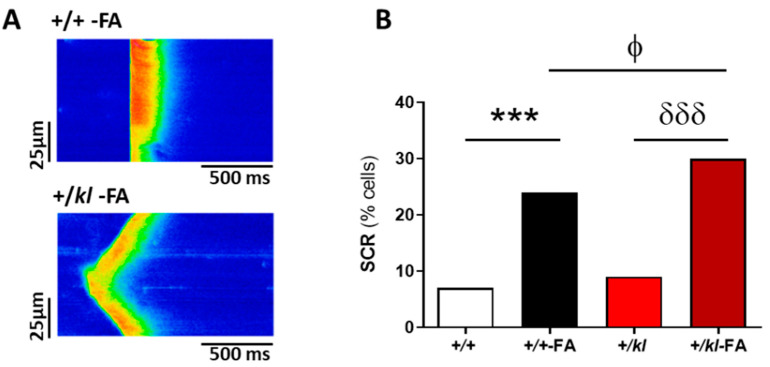
Partial klotho deficiency in AKI conditions augmented the incidence of SCR. (**A**) Representative images of ventricular cardiomyocytes with SCR in the form of an automatic transient (upper panel) or a wave (lower panel) of Ca^2+^ from mice after induction of AKI. (**B**) Percentage of cells that exhibited SCR as the sum of spontaneous Ca^2+^ waves and transients in +/+ (n = 53 cells, N = 5 mice), +/+-FA (n = 82 cells, N = 4 mice), +/*kl* (n = 51 cells, N = 5 mice) and +/*kl*-FA animals (n = 64 cells, N = 3 mice). Data are expressed as a percentage. *** *p* < 0.001 vs. +/+, ^ϕ^ *p* < 0.05 vs. +/+-FA, ^δδδ^ *p* < 0.001 vs. +/*kl*.

We also analysed other forms of spontaneous diastolic Ca^2+^ release (SCR), such as the presence of Ca^2+^ waves or automatic Ca^2+^ transients, in the absence of electrical stimulation. Representative images of both forms of SCR in ventricular cardiomyocytes from AKI-mice are shown in Figure 5A (upper panel: representative of a spontaneous Ca^2+^ transients; bottom panel: representative of a Ca^2+^ wave). The occurrence of the SCR was significantly increased in cardiomyocytes obtained from mice with AKI (*p* < 0.001, Figure 5B). Moreover, SCR was significantly higher in +/*kl*-FA mice when compared to wild-type +/+ mice after AKI (*p* < 0.05, Figure 5B), showing that the deficiency in klotho expression augmented the probability of these aberrant Ca^2+^ events.

### 2.6. Klotho Deficiency Increased the Occurrence of Ca^2+^-Dependent Pro-Arrhythmogenic Events and Following AKI Induction

**Figure 6:**
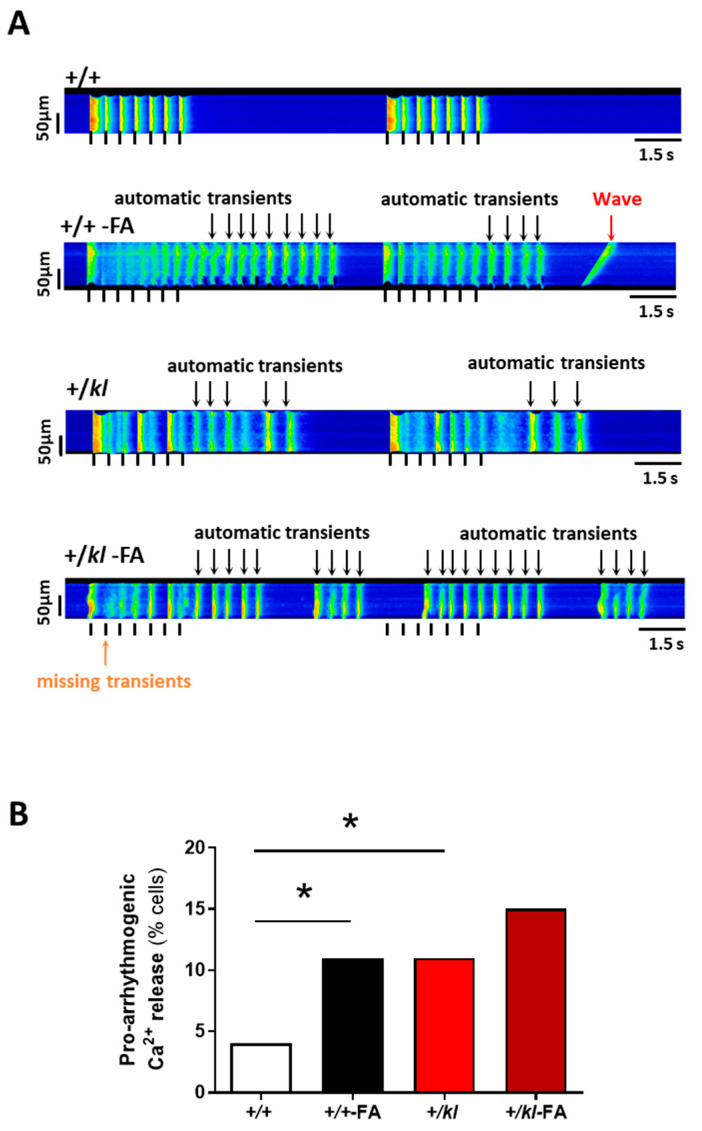
Partial Klotho deficiency increases pro-arrhythmogenic Ca^2+^ events similarly to those observed after AKI. (**A**) Representative line-scan images of cardiomyocytes from each experimental group. Cardiomyocytes were electrically stimulated at 2 Hz in seven-pulse sequences. Black arrows represent automatic Ca^2+^ transient events, the red arrow represents the automatic Ca^2+^ wave and the orange arrow represents missing Ca^2+^ transients (no contraction of the cardiomyocytes during stimulation). (**B**) Quantification of these proarrhythmogenic Ca^2+^ releases following the arrhythmia protocol in cardiomyocytes from +/+ (n = 53 cells, N = 5 mice), +/+-FA (n = 107 cells, N = 4 mice), +/*kl* (n = 51 cells, N = 5 mice) and +/*kl*-FA mice (n = 85 cells, N = 3 mice). Data are expressed as percentage of cells with abnormal behaviour of continuous variables after Fisher test. * *p* < 0.05 vs. +/+.

Finally, to deeply determine whether the partial lack of klotho is involved in the promotion of pro-arrhythmogenic Ca^2+^ events, we applied a cellular arrhythmia protocol during electrical stimulation to isolated ventricular cardiomyocytes. Figure 6A shows representative examples of the arrhythmia protocol in each experimental group. A quantification of the number of cells showing any type of alteration in Ca^2+^ release was performed, considering the presence of pro-arrhythmogenic events such as Ca^2+^ waves (red arrow), automatic Ca^2+^ transients in the absence of electrical stimulation (black arrow) or the absence of Ca^2+^ transients during electrical stimulation (orange arrow). Interestingly, the unique circumstance of klotho expression deficiency induced a significant increase in the frequency of Ca^2+^ release-dependent pro-arrhythmogenic activity at baseline conditions (+/*kl* vs. +/+, *p* < 0.05, Figure 6B) similar to those observed after AKI induction in +/+ mice (*p* < 0.05, Figure 6B). Deficiency in klotho expression together with AKI increased the percentage of pro-arrhythmogenic Ca^2+^ events compared to AKI or klotho deficiency independently.

## 3. Discussion

The main objective of this study was to analyse the presence of alterations in cardiomyocyte function and the occurrence of pro-arrhythmogenic events in a situation of partial deficiency of klotho expression, and whether this circumstance could be aggravated in CRS3.

Here, we performed an experimental nephrotoxic model of FA-induced AKI, in which an abrupt loss of renal function has been shown to have detrimental repercussions on cardiac structure and function [25]. Alterations in mineral metabolism, including those involving klotho deficiency and increased circulating FGF23 levels, have been linked to kidney disease. However, they may also have direct cardiovascular effects in patients in the different stages of CKD and AKI [10]. At an experimental level, it has been recently described that ventricular adult cardiomyocytes show relevant functional damage in the first hours after AKI where kidney klotho expression drops significantly [20]. Here, we demonstrated that klotho deficiency did not significantly modify any parameters of renal damage. In fact, AKI induction provoked renal hypertrophy as well as increased urea, BUN, phosphate and FGF23 levels. These alterations were equally observed in both heterozygous klotho-deficient mice and their wild-type counterparts with AKI, although phosphate and FGF23 levels were not as high in +/*kl*-FA as in +/+-FA, probably as a compensatory mechanism in conditions of klotho deficiency. At the cardiac level, there were not any macroscopic differences indicative of cardiac hypertrophy in any group independently of AKI induction or any deficiency in klotho expression. It is likely that the induction of cardiac hypertrophy needs a more drastic decline in klotho expression. Supporting this, several experimental studies have shown that a total deficiency of klotho (*kl/kl* mice) is able to induce pathological hypertrophy development, which can be attenuated with exogenous klotho administration [26,27,28]. However, despite the absence of morphological cardiac changes—the physiological process that governs the cellular contraction–relaxation cycle—intracellular Ca^2+^ handling can be altered as a primary effect on the cardiomyocytes. We observed that relevant alterations in cardiac Ca^2+^ handling can be detected in the conditions of partial deficiency of klotho expression where an AKI is induced. In fact, these alterations are quite similar to those observed in experimental models of heart failure with alterations in cellular contraction, a decrease in systolic Ca^2+^ release and an increase in the diastolic Ca^2+^ leak [29,30]. Here, we observed a significant decrease in systolic Ca^2+^ release in cardiomyocytes isolated from mice with kidney damage, independently of the genotype of the animals; although mice with reduced klotho expression had higher Tau, which probably means a slower SERCA2a action and Ca^2+^ reuptake velocity into SR, compared to wild-type mice under basal condition. Moreover, when AKI is induced in heterozygous hypomorphic +/*kl* mice, the Ca^2+^ reuptake velocity, which is an indirect measure of SERCA2a activity, compared with heterozygous +/*kl* mice without AKI was significantly decreased probably as a consequence of the reduction in systolic Ca^2+^ release observed in these mice. In uremic conditions, some disturbances in cellular Ca^2+^ metabolism are frequently observed due to the effects of uremic toxins, especially at the renal level, although more studies are needed to elucidate its consequences in the CRS setting as in cardiomyocytes. However, it is logical to think that uremic toxins as phenylacetic acid, with known capacity to block plasma membrane Ca^2+^-ATPase [31], could be also involved in the regulation of SERCA2a activity in AKI conditions. However, further studies are needed to corroborate this possibility.

All the disturbances in Ca^2+^ handling observed in the present study are translated into alterations in contraction with a significantly worsening cellular shortening in those animals with partial klotho depletion with AKI. Thus, the cell shortening of cardiomyocytes from heterozygous hypomorphic AKI-mice was lower than observed in control heterozygous hypomorphic mice. Taken together, partial klotho depletion aggravates cardiomyocyte contraction after AKI, most likely because of the relevant alterations in the systolic Ca^2+^ handling. Moreover, cardiomyocytes isolated from AKI mice had a lower SR-Ca^2+^ load than vehicle-treated mice, demonstrating that AKI induces a decrease in SR Ca^2+^ load. Similar results of decreased SR Ca^2+^ load were obtained in studies carried out in ventricular cardiomyocytes from other experimental models of renal damage such as 5/6 nephrectomy as a model of CKD development [18]. In this sense, the increased Ca^2+^ leak observed during diastole after AKI induction may explain the decrease in the Ca^2+^ load contained in the SR, in turn compromising Ca^2+^ release during electrical stimulation and, therefore, cellular contraction.

It was observed that the frequency of Ca^2+^ sparks was significantly increased in the heterozygous hypomorphic +/*kl* group and was aggravated after AKI induction. This diastolic Ca^2+^ leak is a consequence of an increase in RyR_2_ activity estimated indirectly through the quantification of the frequency of Ca^2+^ sparks [32]. An increase in the occurrence of Ca^2+^ sparks during diastole has been associated with cardiac dysfunction, especially with the predisposition to arrhythmias [33,34], since the Ca^2+^ released can induce the activation of adjacent RyR_2_ producing spontaneous Ca^2+^ waves capable of provoking automatic ventricular contractions [30,35]. Our results showed that AKI itself induces a major incidence of aberrant SCR and pro-arrhythmogenic Ca^2+^ events. Partial klotho depletion predisposed cardiomyocytes to enhance automaticity due to the increased sensitivity of RyR_2_ to Ca^2+^ leak during diastole. Furthermore, when heterozygous klotho-deficient mice suffered AKI, both Ca^2+^ spark frequency and the automaticity activity of cardiomyocytes were even more significantly increased compared to their baseline condition. This aspect has important translational clinical relevance as it may explain why patients are at high risk of cardiac events, specifically fatal arrhythmias following AKI. As shown here, the higher predisposition to fatal arrhythmias could be a consequence of altered intracellular Ca^2+^ handling in their ventricular cardiomyocytes, which is observed only a few hours after AKI.

Previous studies in experimental AKI models have shown that renal and plasma klotho levels decrease dramatically 72 h after FA injection [36]. In addition, AKI patients also show alterations in klotho levels, with some authors reporting a decrease and others an increase in serum klotho levels [20,37,38,39]. These opposite results could be explained by the fact that the relation between kidney function and soluble klotho is not closely linear, depending on the time in which klotho is measured after AKI. At the cardiac level, there is evidence that klotho exerts a cardioprotective role, since its deficit is linked to uremic cardiac remodelling [40] and its exogenous supplementation protects the cardiac function [18,24]. Here, we demonstrated that a partial klotho deficiency that occurs with any evidence of renal disease, could have an added deleterious impact in the setting of AKI. At the clinical level, a decrease in klotho levels may be due to several causes, such as aging and being in the early stages of renal disease, which can remain as a silent disease for many years until it is clinically detected. In the CRS setting, high klotho levels are associated with a lower risk of decline in kidney function [41]. At the same time, lower klotho levels seem to be a potent marker of cardiovascular risk, especially in an aging situation [13].

Finally, current treatments for AKI are unsatisfactory and not directed to avoid cardiac complications. Thus, more studies are needed to elucidate new mechanisms that can be traduced into novel therapeutic targets and subsequently strategies that might significantly reduce cardiac complications in AKI patients. In this sense, the exogenous supplementation of recombinant soluble klotho in +/*kl*-FA animals would be an interesting option to be tested in future studies. Supporting this idea, exogenous klotho supplementation has been recently shown to have a relevant cardioprotective action in CKD [18,22,24] or AKI [23,42]. This novel protective action of klotho supplementation includes the regulation of several intracellular mechanisms as an enhancement in Ca^2+^ mishandling with a reduction in RyR_2_ hyperactivity and reduction of its phosphorylation at Ca^2+^/calmodulin-dependent protein kinase type II (CaMKII) [18,43], together with the prevention of the decrease in the transient outward potassium (K+) current, avoiding the QT interval prolongation [24] at cardiomyocyte level. Similarly, elevation in klotho levels has been demonstrated to the inhibit other ion channels such as the transient receptor potential channel 6 (TRPC6) and transient receptor potential vanilloid type 5 (TRPV5) channels at both cardiac and renal level [44,45,46]. Thus, all these effects on intracellular mechanisms of exogenous klotho supplementation could be preventing the CRS associated with uremic cardiomyopathy. Finally, in addition to experimental studies, future research in humans would be also needed to finally confirm the cardioprotective action of exogenous klotho supplementation in the CRS setting.

In conclusion, we have demonstrated that partial klotho deficiency aggravates the altered cardiac Ca^2+^ handling induced by AKI. This opens the possibility to therapeutic strategies aimed at increasing the bioavailability of klotho as a preventive tool against the occurrence of fatal cardiac events that frequently occur in patients with AKI.

## 4. Materials and Methods

### 4.1. Animal Study

Klotho-hypomorphic (*kl*/*kl*) mice were kindly provided by Dr Kuro-o and bred in our animal facilities. Klotho-deficient mice were generated with transgene integration into the klotho locus, leading to disruption of the 5′-flanking region and the loss of expression as previously described [1]. In origin, this colony was created to obtain independent transgenic founder mice, all of them produced by microinjection of a linearized construct that expresses mouse kl cDNA under the control of the human elongation factor EF-1a promoter, thought to be ubiquitously active at all developmental stages [1]. The resulting transgenic mice were indistinguishable from wild-type mice. They were first mated with +/*kl* mice to obtain transgenic mice carrying the heterozygous *kl* mutation in the F1 generation; these mice were then backcrossed with +/*kl* mice and, in the F2 generation, transgenic mice carrying the *kl*/*kl* homozygous mutation together with +/+ and +/*kl* were obtained and their phenotypes were analysed [1]. We mated +/*kl* mice and obtained all the phenotypes, but for the present study we used heterozygous hypomorphic mice (+/*kl*), and their +/+ littermates of 20–25 g in weight. Animals were group housed in cages at the Experimental Animal Centre, Hospital Universitario 12 de Octubre Madrid, Spain, and were maintained at a controlled and constant temperature (23–25 °C) on a 12 h light/dark cycle with ad libitum access to water and a standard diet. Mice were genotyped by tail DNA and PCR as follows: an initial denaturation at 94 °C for 2 min, followed by 30 cycles of denaturation at 94 °C for 30 s; annealing at 56 °C for 30 s; and extension at 72 °C for 1 min. A common primer (TGGAGATTGGAAGTGGACG) and a wild-type-specific primer (TTAAGGACTCCTGCATCTGC) amplified a 458 bp fragment from the wild-type klotho allele. The common primer and a mutation-specific primer (CAAGGACCAGTTCATCATCG) amplified a 920 bp fragment from the Klotho mutant allele.

### 4.2. Experimental FA-AKI Model

AKI was induced by an FA overdose. Mice were injected intraperitoneally (i.p.) with FA at a dose of 250 mg/kg diluted in 0.3 M sodium bicarbonate (vehicle). Animals were sacrificed 24 h after AKI induction. Different tissues were collected from each animal, such as heart, kidneys and tibia, and blood was collected in heparinised tubes. The blood was centrifuged for 10 min at 2000 rpm to obtain plasma. Hearts and kidneys were collected and kept at −80 °C in a freezer until processed for further analysis.

### 4.3. Analysis of Macroscopic Parameters and Biochemical Assays

To evaluate the possible development of renal and/or cardiac hypertrophy as a consequence of FA-induced AKI, data on BW, KW and HW, as well as TL were obtained after the sacrifice of the animals. Just prior to sacrifice, blood samples from the mice were collected for plasma collection. To study kidney damage, we analysed urea and BUN plasmatic levels (BioAssays Systems, Hayword, CA, USA). In addition, the Phosphate Assay Kit (Abcam, Cambridge, UK) was used to analyse plasmatic levels of phosphate. All assays were used according to the manufacturers’ recommendations. Plasma levels of cFGF23 (C-terminal FGF23, Immunotopics, Inc., San Clemente, CA, USA) were assessed by ELISA kits according to the manufacturer’s instructions.

### 4.4. RNA Isolation and Quantitative Real-Time PCR

Total RNA was isolated from kidney tissue using the RNeasy Mini Kit (Qiagen, Hilden, Germany) according to the manufacturer’s instructions. RNA was reverse transcribed using the cDNA High-Capacity cDNA Reverse Transcription Kit (Applied Biosystems, Foster City, CA, USA). Quantitative RT-PCR was performed using FastStart Essential DNA Green Master (Roche, Basel, Switzerland) in a 10 µL total reaction volume on a LightCycler^®^ 480 II instrument (Roche, Basel, Switzerland) at optimized thermocycling settings. Relative gene expression was normalized to ribosomal *36b4* (RPLP0) as a housekeeping gene, evaluated using the 2^−ΔΔCt^ method and reported as fold change. The sequences (5′–3′) of the primers used were as follows: Klotho-Forward primer TGTGACTTTGCTTGGGGAGTT and reverse primer TCTTCTTGGCTACAACCCCG; KIM-1-Forward primer AGGCGCTGTGGATTCTTATG and reverse primer AAGCAGAAGATGGGCATTGC; NGAL-Forward primer GGCCAGTTCACTCTGGGAAA and reverse primer TGGCGAACTGGTTGTAGTCC; RPLP0-Forward primer AGATGCAGCAGATCCGCAT and reverse primer GTTCTTGCCCATCAGCACC.

### 4.5. Adult Ventricular Cardiomyocytes Isolation

Twenty-four hours after AKI induction by FA injection, the mice were anesthetised and sacrificed with sodium pentobarbital-heparin (100 mg/kg, i.p.). The hearts were rapidly removed and cannulated via the ascending aorta onto a Langendorff perfusion system. A Ca^2+^-free Tyrode’s solution supplemented with EGTA (0.2 mmol/L) was used to retrograde perfuse the hearts over 2–3 min at room temperature. After that, the hearts were digested using a type II collagenase solution (1mg/mL, Worthington, Lakewood, NY, USA) and CaCl_2_ (0.1 mmol/L) for 3 to 5 min. The hearts were then removed from the Langendorff apparatus and ventricles were cut into small pieces to disperse the isolated ventricular cardiomyocytes. Digested hearts were filtered through a 250-µm nylon mesh and centrifuged at room temperature at 300 rpm for 3 min. Finally, the resulting pellet was resuspended in Tyrode’s solution with CaCl_2_ (1 mmol/L) and BSA (2mg/mL). Only electrically excitable, rod-shaped cardiomyocytes with clear cross-striations, which were also Ca^2+^-tolerant in Tyrode solution with increasing Ca^2+^ concentration (from 0.1, 0.5 to 1 mmol/L), were used for imaging studies of intracellular Ca^2+^. Tyrode’s solution contained (in mM): 130 NaCl, 5.4 KCl, 0.4 NaH_2_PO_4_, 0.5 MgCl_2_, 25 HEPES and 22 Glucose. The pH was adjusted to 7.4 with LiOH.

### 4.6. Analysis of Intracellular Ca^2+^ by Confocal Microscopy

Isolated ventricular cardiomyocytes were loaded with the cell-permeant fluorescent calcium dye Fluo-3 AM (5 µmol/L; Invitrogen, Carlsbad, CA, USA) for 30 min at room temperature. Myocytes were field stimulated by two parallel platinum electrodes at 2 Hz. Cell shortening was measured using the difference in cardiomyocyte length between electrical stimulation and the resting period and was expressed as the percentage of the cell’s shortening length. Intracellular Ca^2+^ was imaged by confocal microscopy (Meta Zeiss LSM510, objective 40×, 1.2 NA) at a speed of 1.5 ms/line (1000 lines per image) to record Ca^2+^ transients and Ca^2+^ spark images and at 3 ms/line (10,000 lines per image) for caffeine and arrhythmia protocols. All Ca^2+^ images were corrected for background fluorescence. For Ca^2+^ transients, the fluorescence value (F) was normalised by baseline fluorescence (F_0_) to obtain F/F_0_. The decay time constant of the Ca^2+^ transients, *Tau*, was obtained by adjusting the decay trace of the fluorescence to a single exponential and was used to indirectly estimate SERCA2a function. *K* SERCA2a refers to the SR-dependent fraction of the rate constant of decay of the systolic Ca^2+^ transient and was measured by subtracting the rate constant of decay of the caffeine-evoked transients from that of the systolic Ca^2+^ transients [47]. The Ca^2+^ load of the SR was estimated in intact ventricular myocytes by a quick administration of 10 mmol/L caffeine to deplete Ca^2+^ stores. To quantify spontaneous Ca^2+^ sparks, Fluo-3AM-loaded quiescent myocytes were maintained without electrical stimulation and Ca^2+^ spark regions were determined as the specific sites where the fluorescence signal briefly increased by at least four times the standard deviation of the fluorescence. Total spark-mediated Ca^2+^ leak was calculated by multiplying spark frequency × peak (F/F_0_) × duration (FDHM) × width (FWHM). SCR was quantified by the percentage occurrence of Ca^2+^ waves or automatic Ca^2+^ transients without electrical stimulation. Two seven-pulse cycles of a 2 Hz electrical stimulation field with a rest period were then applied to record pro-arrhythmogenic activity. Arrhythmic Ca^2+^ events were considered to be any spontaneous abnormal release of Ca^2+^ as waves, missing transients or automatic contractions during the protocol. All Ca^2+^ images were recorded using the following recording solution containing (in mM): 140 NaCl, 4 KCl, 1.8 CaCl_2_, 1.1 MgCl_2_, 10 HEPES and 11 Glucose with pH adjusted to 7.4 using LiOH. All Ca^2+^ images were processed and analysed using home-made routines with IDL (Research Systems, Boulder, CO, USA) and Image J (National Institutes of Health, Bethesda, MD, USA) software programs.

### 4.7. Statistical Analysis

All collected data were first tested for normality using the Shapiro–Wilk test. Statistical significance was accomplished by applying an analysis of variance (ANOVA) with a Newman–Keuls multiple comparison test for normally distributed data or the Kruskal–Wallis test with Dunn’s correction for non-normally distributed data. The *χ*^2^ test was used when appropriate. Group measurements were expressed as mean ± SEM. Values of *p* < 0.05 were considered statistically significant. All analyses were performed using an OriginPro 9.0 (OriginLab, Northampton, MA, USA) or GraphPad Prism v6.0 (GraphPad Software Inc., San Diego, CA, USA).

## Figures and Tables

**Table 1:** Macroscopic parameters in *+/+* and *+/kl* mice after AKI.

Parameters	*+/+*	*+/+*-FA	*+/kl*	*+/kl*-FA
BW (g)	24.0 ± 0.4	22.5 ± 0.3	24.8 ± 0.8	23.1 ± 0.8
TL (mm)	17.1 ± 0.2	17.7 ± 0.5	17.1 ± 0.2	17.2 ± 0.7
KW (g)	158.2 ± 8.4	202.9 ± 14.8 *	163.1 ± 8.6	216.1 ± 17.1 ^δδ^
KW/BW (mg/g)	6.8 ± 0.2	9.0 ± 0.6 ***	6.6 ± 0.2	9.3 ± 0.5 ^δδδ^
KW/TL (mg/mm)	9.6 ± 0.3	11.6 ± 1.0	9.6 ± 0.5	12.8 ± 1.4 ^δ^
HW (g)	172.9 ± 10.6	187.0 ± 8.6	182.8 ± 12.4	180.3 ± 17.7
HW/BW (mg/g)	7.2 ± 0.4	8.3 ± 0.3	7.4 ± 0.5	7.7 ± 0.6
HW/TL (mg/mm)	10.1 ± 0.6	10.6 ± 0.5	10.7 ± 0.7	10.7 ± 1.3

Data from 6–10 animals for macroscopic parameter per experimental group are reported as mean ± SEM. BW: body weight; TL: tibia length; HW: heart weight; KW: kidney weight. * *p* < 0.05, *** *p* < 0.001 vs. *+/+*; ^δ^ *p* < 0.05, ^δδ^ *p* < 0.01, ^δδδ^ *p* < 0.001 vs. *+/kl*.

**Table 2:** Biochemical plasma parameters in *+/+* and *+/kl* mice after AKI.

Parameters	+/+	+/+-FA	+/*kl*	+/*kl*-FA
Urea (mg/dL)	33.8 ± 1.8	389.0 ± 27.3 ***	40.9 ± 3.4	418.2 ± 45.5 ^δδδ^
BUN (mg/dL)	15.8 ± 0.8	181.8 ± 12.8 ***	19.1 ± 1.6	195.4 ± 21.3 ^δδδ^
Phosphates (mg/mL)	12.8 ± 0.5	33.9 ± 3.3 ***	9.6 ± 0.5	23.7 ± 2.5 ^ϕϕϕ, δδδ^
FGF23 (pg/mL)	237 ± 21	13190 ± 1820 ***	250 ± 18	8648 ± 2560 ^ϕ, δδδ^

Data from 6–10 animals for biochemical renal parameters per experimental group are reported as mean ± SEM. BUN: blood urea nitrogen. FGF23: fibroblast growth factor 23. *** *p* < 0.001 vs. *+/+*; ^ϕ^ *p* < 0.05, ^ϕϕϕ^ *p* < 0.001 vs. *+/+*-FA, ^δδδ^ *p* < 0.001 vs. +/*kl*.

## Data Availability

Not applicable.

## Supplementary Materials

The following supporting information can be downloaded at: https://www.mdpi.com/article/10.3390/ijms24021322/s1.
