# Supplementary material for: Partial Genetic Deletion of Klotho Aggravates Cardiac Calcium Mishandling in Acute Kidney Injury
Source: Int J Mol Sci. 2023 Jan 10;24(2):1322. doi: 10.3390/ijms24021322 (PMC9867237; doi:10.3390/ijms24021322)
Supplement: Supplementary file 1 [file ijms-24-01322-s001.zip › ijms-2086353-supplementary.pdf]

Title: Partial Genetic Deletion of Klotho Aggravates Cardiac Calcium Mishandling in Acute Kidney Injury

Authors: Laura González-Lafuente, José Alberto Navarro-García, Ángela Valero-Almazán, Elena Rodríguez-Sánchez, Sara Vázquez-Sánchez, Elisa Mercado-García, Patricia Pineros, Jonay Poveda, María Fernández-Velasco, Makoto Kuro-o, Luis M. Ruilope and Gema Ruiz-Hurtado

Supplemental Material

Supplementary Figures

Supplemental Figure S1. Biomarkers of kidney injury. Renal mRNA expression of (**A**) kidney injury molecule-1 (KIM-1), (**B**) neutrophil gelatinase-associated lipocalin (NGAL) and (**C**) Klotho, obtained from wild-type +/+ (N = 5), +/+-FA (N = 5), +/*kl* (N = 5) and +/*kl*-FA animals (N = 5). Data are shown as mean ± SEM. ** $p < 0.01$, *** $p < 0.001$ *vs.* +/+; $^{\delta\delta} p < 0.01$, $^{\delta\delta\delta} p < 0.001$ vs. +/*kl*; $^{\Phi\Phi\Phi} p < 0.001$ *vs.* +/+-FA.

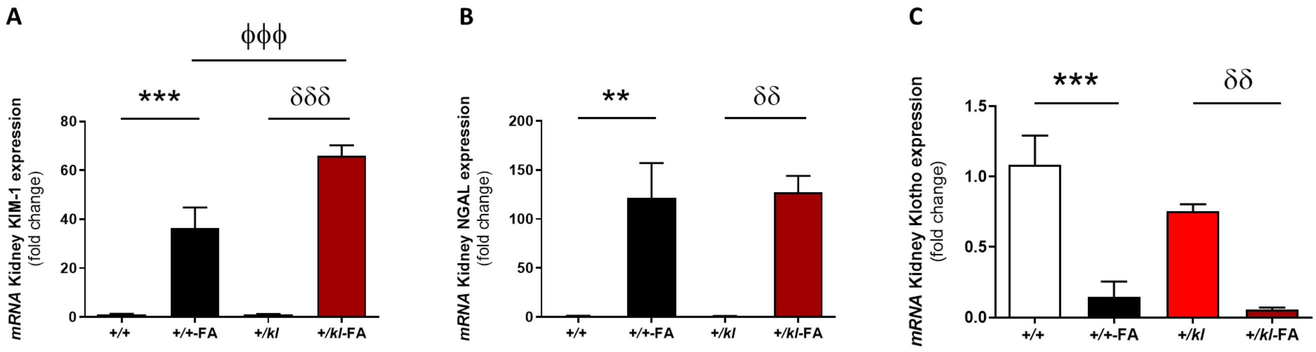

Supplemental Figure S2. *K* SERCA2a in cardiomyocytes from +/+ (n = 21 cells, N = 5 mice), +/+-FA (n = 20 cells, N = 4 mice), +/kl (n = 21 cells, N = 5 mice) and +/kl-FA animals (n = 18 cells, N = 3 mice). Data are expressed as mean ± SEM. $^{\delta}$ $p < 0.001$ *vs.* +/*kl*.

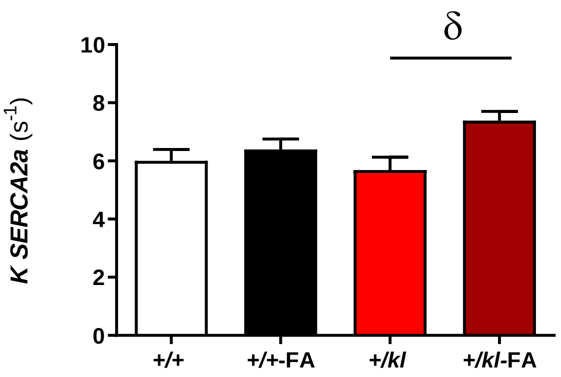

Supplementary Table

Supplementary Table S1. Ca^{2+} spark characteristics in cardiomyocytes from +/+ and +/*kl* mice after AKI. FDHM: full width at half maximum; FDHM: full duration at half maximum.

**Parameters**	**+/+**	**+/+-FA**	**+/*kl***	**+/*kl*-FA**
Peak (F/F_0)	1.33 ± 0.02	1.25 ± 0.01***	1.32 ± 0.01	1.23 ± 0.01$^{\delta\delta\delta}$
FWHM (μm)	3.07 ± 0.09	2.33 ± 0.08***	3.27 ± 0.07	2.33 ± 0.07$^{\delta\delta\delta}$
FDHM (ms)	32.37 ± 1.23	30.21 ± 0.88	39.22 ± 1.74***	32.02 ± 0.98$^{\delta\delta\delta}$

*** $p < 0.001$ *vs.* +/+; $^{\delta\delta\delta}$ $p < 0.001$ *vs.* +/*kl*.
